# Supplementary material for: Pilot randomised controlled trial of the Risk Acceptance Ladder (RAL) as a tool for targeting health communications
Source: PLoS One. 2021 Nov 15;16(11):e0259949. doi: 10.1371/journal.pone.0259949 (PMC8592458; doi:10.1371/journal.pone.0259949)
Supplement: S1 Appendix — (DOCX) [file pone.0259949.s001.docx]

**S1 Appendix**

**1. Home page**

**Welcome text**

We are a team of researchers from University College London studying reasons why people engage in activities that affect their health and how some people might be encouraged to adopt more healthy lifestyles.

We would be extremely grateful if you could help with this study by completing a short questionnaire. We think it should take no more than about 10 minutes of your time.

Thank you very much for your help.

**Explanation of study**

You will be asked a few questions including questions on smoking, alcohol consumption, physical activity, and fruit and vegetable consumption. Then you might also be provided with some information about one of these activities. The survey will last approximately 10 minutes.

You are free to choose whether or not to take part in this study and of course you can stop at any time.

To take part, you should be at least **18 years** of age and a **UK resident.**

As a thank you for helping with our research we would like to offer you the option to enter into a prize draw to win **one of four £50 prizes**. You will only be entered into the prize draw if you complete the survey.

If you would like to be entered into the prize draw please enter your email address below

<**space to enter email address**>

All personal information, including your email address, will be held in strict confidence. After the prize draw, your email address will be deleted. The results of the study may be published for scientific purposes but publications will not contain any personal information that could identify you. This study has been approved by University College London’s Committee on the Ethics of Non-NHS Human Research [Project ID: 6692/001].

If you have any questions or would like additional information about the study, your rights as a participant, or wish to receive a summary of the overall results of the project please contact the research team.

**2. Behavioural questionnaire (100w)**

Here is a short questionnaire including questions about your lifestyle.

Please make sure you answer all the questions.

|  | |
| --- | --- |
| Q.1 Gender | □ Male  □ Female |
| Q.2 Age | Please enter your age in years |
| Q.3 Ethnicity | Please indicate your ethnicity  □ White - British/Irish/Other White  □ Black - African/Caribbean/Other Black  □ Asian - Indian/Pakistani/Bangladeshi/Chinese/Other Asian  □ Mixed - White/Black White/Asian Other Mixed  □ Other (please specify): _________________________ |

|  | |
| --- | --- |
| Q.4 Importance of health | How much does your health matter to you?  □ Very much  □ Quite a bit  □ Somewhat  □ A little  □ Not at all |

|  | |
| --- | --- |
| Q.5 Smoking | Do you smoke cigarettes at all nowadays?  □ Yes  □ No |
| Q.6 Alcohol | Do you drink alcohol every day or almost every day?  □ Yes  □ No |
| Q.7 Physical activity | Do you make sure that you walk or do other moderate physical activity for at least 30 minutes every day?  □ Yes  □ No |
| Q.8 Fruit and Vegetables | Do you make sure that you eat at least 5 portions of fruit and vegetables each day?  □ Yes  □ No |

**3.a. Focus on smoking – instructions (100w)**

The rest of this survey will focus on smoking.

Please answer the question below.

**Which best describes your relationship with smoking?**

1. **I am trying to quit smoking**
2. **I have made a definite plan to quit smoking soon**
3. **I have no immediate plans to quit smoking**

**4.a What might it take?**

Now we would like you to think carefully about what it might take for you to stop smoking.

Please read through the statements below, and select the one statement that you think most closely applies to you.

**I would stop smoking now but…**

|  |  |
| --- | --- |
| 1. I have never heard that smoking was harmful or risky |  |
| 1. I heard that smoking is risky but never fully understood what the problem was |  |
| 1. I understand what people are saying about the risks of smoking, but I don’t believe it |  |
| 1. I accept that smoking is risky but don’t care enough to do anything about it |  |
| 1. I think it is worth quitting smoking but it is not a priority at the moment |  |
| 1. There are too many people around me who smoke |  |
| 1. Things going on in my life at the moment would make it too difficult |  |
| 1. I don’t think I can quit smoking because I don’t have the self-control |  |
| 1. I don’t know the best way of doing it |  |
| 1. None of the above   Please specify ……………… |  |

How easy was it to choose just one answer?

□ Very easy

□ Quite easy

□ Not very easy

□ Not at all easy

**3.b. Focus on Alcohol – instructions (100w)**

The rest of this survey will focus on alcohol.

Please answer the question below.

**What best describes your relationship with alcohol?**

1. **I am trying to cut down on my drinking**
2. **I have made a definite plan to cut down on my drinking soon**
3. **I have no immediate plans to cut down on my drinking**

**4.b What might it take?**

Now we would like you to think carefully about what it might take for you to cut down the amount you drink.

Please read through the statements below, and select the one statement that most closely applies to you.

**I would cut down on the amount that I drink but…**

|  |  |
| --- | --- |
| 1. I have not heard anything to suggest that the amount that I drink is harmful |  |
| 1. I have heard that the amount I drink is risky but I have not really understood why |  |
| 1. I understand what people are saying about the risks of the amount I drink, but I don’t believe it |  |
| 1. I accept that drinking too much is risky but don’t care enough to do anything about it |  |
| 1. I think it is worth cutting down the amount I drink, but it is not a priority at the moment |  |
| 1. I don’t think I can cut down the amount I drink because things in my social world make it too difficult |  |
| 1. I don’t think I can cut down the amount I drink because things going on in my life make it too difficult |  |
| 1. I don’t think I can cut down the amount I drink because I don’t have the self-control |  |
| 1. I want to cut down the amount I drink, but I don’t know the best way to do it |  |
| 1. None of the above   Please specify…………… |  |

How easy was it to choose just one answer?

□ Very easy

□ Quite easy

□ Not very easy

□ Not at all easy

**3.c Focus on physical activity – instructions (100w)**

The rest of this survey will focus on physical activity.

Please answer the question below.

**Which best describes your relationship with physical activity?**

1. **I am trying to become more physically active**
2. **I have made a definite plan to become more physically active soon**
3. **I have no immediate plans to become more physically active**

**4.c What might it take?**

Now we would like you to think carefully about what it might take for you to become more physically active.

Please read through the statements below, and select the one statement that most closely applies to you.

**I would increase the amount that I am active but…**

|  |  |
| --- | --- |
| 1. I never heard that being inactive was harmful or risky |  |
| 1. I heard that being inactive is risky but never fully understood what the problem was |  |
| 1. I understand what people are saying about the risks of being too inactive, but I don’t believe it |  |
| 1. I accept that being inactive is risky but don’t care enough to do anything about it |  |
| 1. I think it is worth becoming more active, but it is not a priority at the moment |  |
| 1. I don’t think I can become more active because things in my social world make it too difficult |  |
| 1. I don’t think I can become more active because things going on in my life make it too difficult |  |
| 1. I don’t think I can become more active because I don’t have the self-control |  |
| 1. I want to become more active, but I don’t know how best to do it |  |
| 1. None of the above   Please specify………….. |  |

How easy was it to make your choice?

□ Very easy

□ Quite easy

□ Not very easy

□ Not at all easy

**3.d. Focus on fruit and veg – instructions (100w)**

The rest of this survey will focus on diet.

Please answer the question below.

**Which best describes your relationship with healthy eating?**

1. **I am seriously trying to eat more fruit and vegetables**
2. **I have made a definite plan to eat more fruit and vegetables soon**
3. **I have no immediate plans to eat more fruit and vegetables**

**4.a What might it take?**

Now we would like you to think carefully about what it might take for you to eat more fruit and vegetables.

Please read through the statements below, and select the one statement that most closely applies to you.

**I would eat more fruit and vegetables but…**

|  |  |
| --- | --- |
| 1. I have not heard that eating not enough fruits and vegetables was harmful or risky |  |
| 1. I have heard that eating a poor diet is risky but never fully understood what the problem is |  |
| 1. I understand what people are saying about the risks of eating a poor diet, but I don’t believe it |  |
| 1. I accept that eating a poor diet is risky but don’t care enough to do anything about it |  |
| 1. I think it is worth eating more healthily, but it is not a priority at the moment |  |
| 1. I don’t think I can eat more healthily because things in my social world make it too difficult |  |
| 1. I don’t think I can eat more healthily because things going on in my life make it too difficult |  |
| 1. I don’t think I can eat more healthily because I don’t have the self-control |  |
| 1. I want to eat more healthily, but I don’t know how best to do it |  |
| 1. None of the above   Please specify……………. |  |

How easy was it to make your choice?

□ Very easy

□ Quite easy

□ Not very easy

□ Not at all easy

**5. Something to help you on your way**

Thank you very much for answering those questions. Based on your answers we have selected some information that we think might interest you.

| 1. **Smoking** | **A** | Not everyone knows quite how harmful smoking is. If they do not stop, smokers die an average of 10 years earlier than non-smokers and smokers suffer from diseases of old age much earlier than non-smokers. There is no safe level of smoking. People who do not smoke every day still suffer a huge increase in risk, particularly from heart disease.  Stopping smoking at any age leads to a longer, happier, healthier life. |
| --- | --- | --- |
|  | **B** | It is not always easy to fully understand the risks from something like smoking. Some people think that perhaps it is similar to eating butter or too much salt. Unfortunately it is much worse than that. Being a smoker carries about the same risk as being morbidly obese (BMI>40). By the age of 40, smokers typically look and feel about 10 years older than their true age.  Stopping smoking in ones 20s or 30s can reverse this. |
|  | **C** | Some people find it hard to believe that smoking cigarettes can have such a huge effect on their health, especially if they only smoke a few cigarettes a day. The thing to remember is that the concentrations of cancer-causing chemicals in cigarette smoke are enormous – and would never be allowed in any other product. Exposure to these chemicals in the most sensitive and delicate part of the body, the lungs, causes serious damage to the cells.  Stopping smoking completely is the only way to reverse this damage. |
|  | **D** | It’s easy to put the harm caused by smoking out of one’s mind and not feel overly worried about it. It is only when we get a health scare that we really start to worry – but by then it could be too late. One of the worst diseases caused by smoking is called Chronic Obstructive Pulmonary Disease. The lungs stop working properly and smokers end up not able to breathe – imagine your whole life breathing through a tiny straw and you will have a sense of what it is like.  Stopping smoking can put your mind at rest that the chances of this happening to you are every low indeed. |
|  | **E** | It’s easy to put off the decision to stop smoking – there is always another day. There is a big problem with this. After having smoked for a while, every day smokers carry on costs an average of 6 hours of life! And every day of smoking avoided gains those 6 hours back.  So there is no time like the present – stopping smoking really is urgent. |
|  | **F** | It can be hard to stop smoking when people around you smoke – there are just too many temptations. But people do manage it every day and if one person in a group stops this can prompt others to stop.  Stopping is incredibly important, whatever the obstacles and there is lots of good advice available on how to cope with others smoking when one is trying to stop. |
|  | **G** | It is easy to think that now is not the right time to stop because there is too much going on in one’s life. In fact research has shown that smoking does not help with stress – it actually makes it worse, and that the chances of success at stopping are just as good whenever you try.  Stopping smoking actually helps with stress and there is lots of good advice available for how to deal with life’s pressures without smoking. |
|  | **H** | Many people think they do not have the self-control needed to stop smoking. But it does not have to be like that. There are lots of things one can do to make it so much easier than it used to be. And in fact, how confident you are in your ability to stop has very little bearing on whether you will succeed.  When smokers stop they feel a huge sense of achievement and there is no reason why you could not be one of them. The one thing that guarantees failure is not trying. |
|  | **I** | Stopping smoking can be daunting when one does not know the best way to do it, but there is lots of excellent advice available – based on literally hundreds of studies. The key thing is to make that first step and to follow the advice.  The first step is to resolve to do something – whether it’s stopping today, deciding to stop soon or even just trying to cut down. One can jump in the deep end or just put a toe in the water! |
|  | **J** | If you are ready to stop smoking now – this is a great time to give it a go. |

| 1. **Alcohol** | **A** | Not everyone knows about how harmful it is to drink too much. There guidelines say that men should drink no more than 3 ‘units’ per day and women should drink no more than 2. Even at these levels, drinking increases the risk of cancer and heart disease.  Cutting down to below the recommended levels could be a life saver. |
| --- | --- | --- |
|  | **B** | Understanding why it’s important not to drink too much is a first step to doing something to protect your health. Even at quite low levels, alcohol increases the risk of breast cancer and other cancers by damaging the DNA in your cells. It also increases blood pressure and increases the risk of heart disease. A big problem is that there is no way of knowing who is susceptible – it could be anyone.  It is so much safer to make sure that one stays below the recommended alcohol limits. |
|  | **C** | It may seem hard to believe that getting drunk once or twice a month, or drinking more than the recommended amount, can be so bad for your health. But the guidelines are based on decades of careful research and they have been written to help you make an informed choice about how much you drink. That is because, even quite low levels of drinking increase the risk of cancer and heart disease.  It is so much safer to make sure that one stays below the recommended alcohol limits. |
|  | **D** | It is easy to put the risks from drinking too much out of one’s mind. Cutting down is a long way down the priority list compared with enjoying the effects of alcohol. The trouble is that things have a way of catching up with us and we end up regretting our mistakes when it is too late.  It is worth at least having a look at how one can enjoy life as much or more while drinking less. |
|  | **E** | It is so easy to put off doing something about one’s drinking till another day. The damage from drinking builds up over time and the sooner one can get it down to recommended level the lower the risk of serious disease or something bad happening when intoxicated.  Today is always a good day to make a start at getting drinking down to safe levels. |
|  | **F** | Sometimes it can be hard to cut down one’s drinking when one has an active social life. But there are many ways of doing it and having a good time – or even a better time! In fact, studies have shown that the other people we are with tend to drink less than we think they do – so many of our friends are already keeping within the guidelines.  It’s worth having a look at the excellent advice available on how to socialise without drinking too much. |
|  | **G** | Cutting down on one’s drinking can be hard when there is a lot going on in our lives and we use drinking to help us unwind or cope. The problem is that of course the drinking doesn’t solve problems, it just helps us to forget them for a while and they have a habit of coming back to bite us even harder.  It is worth having a look at all the excellent advice available on how to cope with life’s difficulties without drinking too much. |
|  | **H** | Many people think they don’t have the self-control needed to cut down on their drinking. But there is lots of good advice available to make it so much easier. And we often have a lot more self-control than we think we have.  It is worth having a look at all the excellent advice available on how to cut down on drinking without it being a struggle. |
|  | **I** | Sometimes it’s hard to know where to start in cutting down one’s drinking. But that doesn’t matter. The important thing is that one can start at any time and then start learning from what happens. And decades of research has shown us the kinds of things that are likely to help.  It is worth having a look at all the excellent advice available on how to cut down on drinking without it being a struggle. |
|  | **J** | If you are ready to cut down on your drinking now – this is a great time to make a start. |

| **3. Physical Activity** | **A** | Not everyone knows quite how harmful leading an inactive life is. Over 3million deaths each year worldwide can be attributed to people not getting enough physical activity. The guidelines state that people should aim for at least 150 minutes of moderate physical activity per week.  By becoming more active you will reduce your risk of heart disease by up to 35% and your risk of type II diabetes by up to 50%. |
| --- | --- | --- |
|  | **B** | It can be difficult to understand the beneficial effect that physical activity has on your health. Physical activity improves the condition of your heart and blood vessels which lowers your blood pressure. Extensive research has shown that not getting enough physical activity, or even sitting for too long, puts you at an increased risk of serious conditions such as type II diabetes or heart disease.  Simply by meeting the recommended guidelines of 150 minutes of physical activity per week, you can lower your risk of premature death by up to 30%. |
|  | **C** | Some people find it hard to believe that by simply becoming more active they can lower their risk of premature death by up to 30%.  Years of research has found that increasing levels of physical activity decreases the risk of illnesses such as heart disease, type II diabetes and even some cancers.  Exercise is described as the miracle cure for good reason, increasing your levels of physical activity could be a life saver. |
|  | **D** | It is easy to put the importance of physical activity out of one’s mind. Unfortunately, a lifetime of inactivity does tend to catch up with us. Putting off becoming more active could result in a number of unpleasant illnesses including heart disease or type II diabetes which may not concern you now, but might in the future.  Anyone can experience the benefits of becoming more active and the sooner you start the better. |
|  | **E** | It is easy to put off the decision to become more active. However, it is important to remember that the effects of inactivity add up over a number of years resulting in poor health. Becoming more active now not only reduces the risk of illnesses such as heart disease, but also has more immediate benefits such as improved mood.  You should aim to achieve 150 minutes of physical activity such as brisk walking per week, but any amount of activity is better than no activity so start by doing what you can and aim to increase this over time. |
|  | **F** | Sometimes it can feel as if one’s social life gets in the way of becoming more active, but there are actually ways of making physical activity social. Getting active with a friend or joining a club can make it easier to find the time, or give you the extra motivation you need to get fit.  It is worth having a look at how you can incorporate getting more active into your social life. |
|  | **G** | Becoming more active can feel difficult when there is a lot going on in one’s life. However, becoming more active can actually lower stress levels, increase energy levels and can even lead to improved mood.  It is worth having a look at some of the excellent help available on how to become more active, and finding out for yourself how you can benefit from increasing your activity levels. |
|  | **H** | Some people feel that they cannot increase their levels of physical activity because they don’t have the self-control, but this does not have to be the case. There are so many resources out there that make increasing levels of physical activity as easy as possible. Also, we often have a lot more self-control than we think we have.  It is worth looking at some of the excellent advice available to you and giving it a go. |
|  | **I** | Sometimes it can be difficult to know where to start in increasing one’s physical activity levels.  Although guidelines advise 150 minutes of moderate physical activity per week, any amount of activity is better than no activity. Start off by doing what you can, and aim to increase your activity levels over time.  It is worth looking at some of the excellent advice available on simple ways to increase your activity levels. |
|  | **J** | If you are ready to increase your levels of physical activity – this is a great time to give it a go. |

| **4. Fruit and Veg** | **A** | Not everyone knows how harmful eating too little fruit and veg can be. The guidelines say that people should eat at least 5 portions of fruit and veg every day. If everyone met this target 42,000 deaths could be avoided in the UK each year.  Eating the recommended amount of fruit and veg will reduce your risk of heart disease, stroke and even some cancers. |
| --- | --- | --- |
|  | **B** | It is not always easy to understand why eating a diet high in fruit and veg is so beneficial to your health. Fruit and vegetables are high in fibre- a diet high in fibre can reduce your risk of developing bowel cancer. Fruit and vegetables also provide many of the essential vitamins and minerals necessary for the body to function properly.  By increasing the amount of fruit and vegetables that you eat you will reduce your risk of heart disease, stroke and even some cancers. |
|  | **C** | Some people find the benefits of eating a diet rich in fruit and vegetables hard to believe. Rigorous scientific research has found that eating a diet high in fruit and veg can help reduce your risk of serious health conditions such as heart disease, stroke, and even some cancers. It is also estimated that 42,000 deaths could be avoided in the UK each year if people increased their fruit and veg intake to the recommended 5 a day.  By making small changes to your diet, you could make huge improvements to your health. |
|  | **D** | It is easy to put the benefits of eating a diet high in fruit and veg to the back of one’s mind. However, it is important to remember that over a number of years, eating a diet low in fruit and veg increases one’s chances of developing heart disease, stroke and even some cancers.  It is so much better to eat more fruit and vegetables now than to suffer the consequences in the future. |
|  | **E** | It is easy to put off eating more fruit and veg. Sometimes the consequences of eating too little fruit and veg may not seem obvious or immediate, but unfortunately these things tend to catch up with us. Over a number of years, eating a diet low in fruit and veg increases one’s chances of developing heart disease, stroke and even some cancers.  Improving your diet now is one simple way to reduce your risk of experiencing poor health in the future. |
|  | **F** | Sometimes it can feel as if one’s social life gets in the way of eating more healthily. But an active social life does not have to mean a diet low in fruit and veg.  It is important to think in advance about how you will get your 5 a day. If you eat out a lot, try swapping unhealthier side dishes such as chips for vegetables or a salad. Or try making your new healthy diet social by involving some of your friends.  It is worth having a look at the excellent advice available on simple ways to increase the amount of fruit and veg that you eat. |
|  | **G** | Changing the way we eat can feel difficult when there is a lot going on in our lives. However, eating more fruit and veg doesn’t need to be hard work. Using a mobile app can help you stay on track, and using a meal planner can take some of the effort out of working out how to fit in five portions of fruit and veg each day.  Research has even shown that people who eat more fruit and veg score higher in measures of mental wellbeing, so why not give it a go. |
|  | **H** | Some people feel that they do not have the self-control needed to increase the amount of fruit and veg that they eat. But there are lots of things that can be done to make eating more fruit and veg simple.  It is worth having a look at the excellent advice available on how to increase the amount of fruit and veg that you eat. Give it a go, you might surprise yourself! |
|  | **I** | Sometimes it can be difficult to know where to start when one wants to eat more fruit and veg.  Although guidelines recommend 5 portions of fruit and veg every day, it may seem like a daunting challenge to change ones diet overnight. If this is the case, it may be easier to gradually add a variety of fruit and veg over a number of days or weeks.  It is worth having a look at the excellent advice available on how to add more fruit and veg into your diet. |
|  | **J** | If you are ready to start eating more fruit and veg – this is a great time to give it a go. |

**6. Interested in finding out more?**

*Thank you for using our website and answering our questions. We hope you found it interesting. We have done the research to find what we think is the best website right now for expert advice on how to (program chooses from stop smoking, cut down your drinking, get more exercise, eat more healthily depending on the focus).*

*Click on the link below and this will take you to that website. Otherwise you can simply close this page in your browser to finish the session.*

**6.a Smoking**

For expert advice on quitting smoking, **click** on the link below…

Websites:


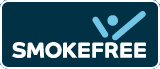


<http://www.nhs.uk/smokefree>

**6.b Alcohol**

For expert advice on how to cut down on your drinking click on the link below …

We need to find a good website – probably Down Your Drink

<http://www.downyourdrink.org.uk/>


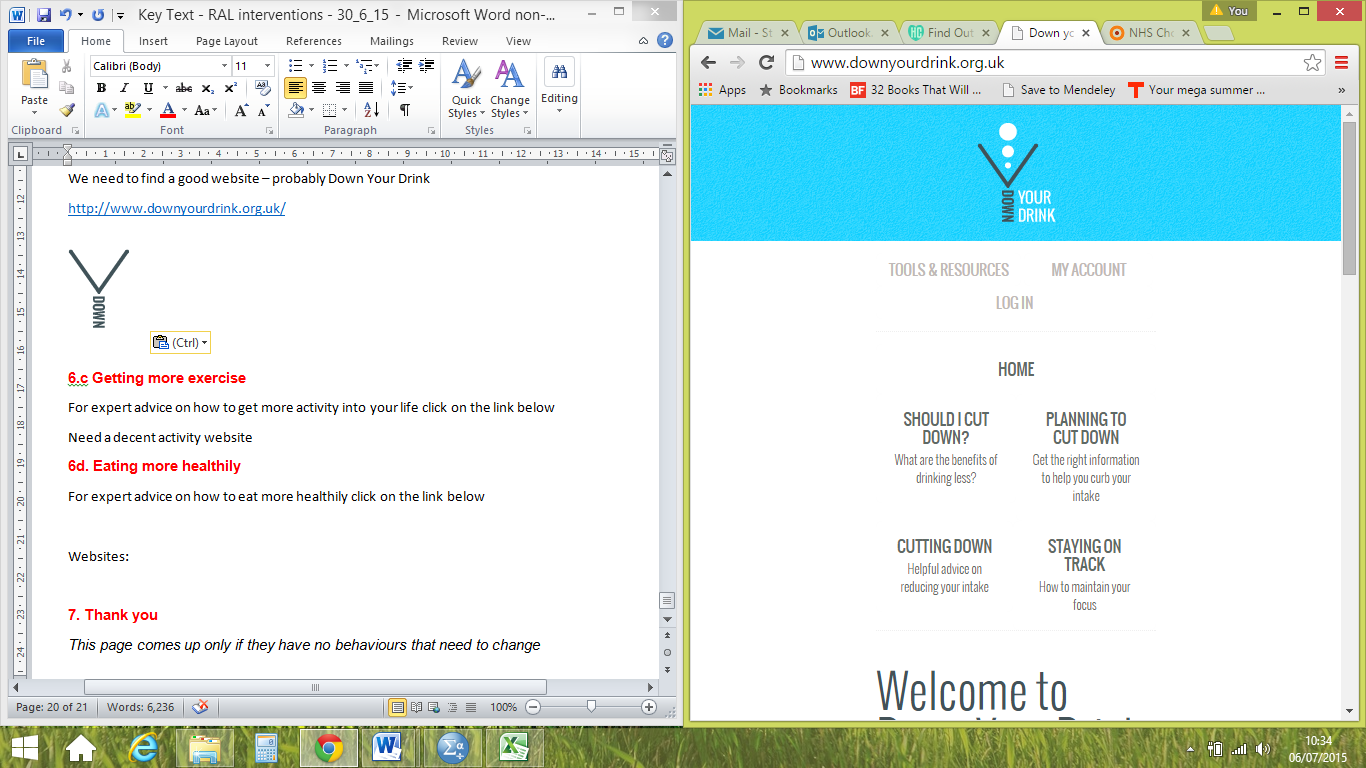


**6.c Becoming more active**

For expert advice on how to get more activity into your life click on the link below

<http://www.nhs.uk/livewell/fitness/Pages/Fitnesshome.aspx>


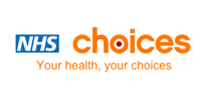


**6d. Eating more healthily**

For expert advice on how to eat more healthily click on the link below

<http://www.nhs.uk/Livewell/5ADAY/Pages/5ADAYhome.aspx>


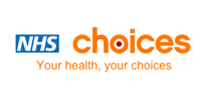


**7. Thank you**

*This page comes up only if they have no behaviours that need to change*

Thank you for taking part in this survey. It looks like you are already doing lots of the things that can help to keep you healthy.

Your responses have been recorded, and will help us to understand the importance of health to different people.
