# Supplementary material for: Pilot randomised controlled trial of the Risk Acceptance Ladder (RAL) as a tool for targeting health communications
Source: PLoS One. 2021 Nov 15;16(11):e0259949. doi: 10.1371/journal.pone.0259949 (PMC8592458; doi:10.1371/journal.pone.0259949)
Supplement: Attachment — Submitted filename: Response to Reviewers_FINAL.docx [file pone.0259949.s002.docx]

**Response to Reviewers**

**Editor**

Please ensure that your manuscript meets PLOS ONE's style requirements, including those for file naming.

*We have now ensured the manuscript is compliant with PLOS ONE’s style requirements (e.g. all figures are uploaded as separate files, the manuscript is double spaced, supplementary files have been labelled according to the style guidelines).*

Thank you for stating the following in the Competing Interests section: "I have read the journal's policy and the authors of this manuscript have the following competing interests: OP and CS report no conflicts of interest. RW has undertaken research and consultancy for and receives travel funds and hospitality from manufacturers of smoking cessation medications (Pfizer, GlaxoSmithKline and Johnson and Johnson). LS has received a research grant and honoraria for a talk and travel expenses from manufacturers of smoking cessation medications (Pfizer and Johnson & Johnson)." Please confirm that this does not alter your adherence to all PLOS ONE policies on sharing data and materials, by including the following statement: "This does not alter our adherence to PLOS ONE policies on sharing data and materials.” (as detailed online in our guide for authors http://journals.plos.org/plosone/s/competing-interests). If there are restrictions on sharing of data and/or materials, please state these. Please note that we cannot proceed with consideration of your article until this information has been declared. Please include your updated Competing Interests statement in your cover letter; we will change the online submission form on your behalf.

*We have confirmed that this does not alter our adherence to PLOS ONE’s data sharing policies by including an updated Competing Interest statement in our cover letter.*

Please include captions for your Supporting Information files at the end of your manuscript, and update any in-text citations to match accordingly.

*This has now been addressed.*

Write the Abstract according to the following algorithm: first two or three sentences indicate the relevance of the topic; the aim and object of the study; the methodology (methods) of the study (for theoretical studies – its theoretical basis) are described; the obtained results and their practical value are characterized. Dedicate most of the Abstract to the result. The volume of the Abstract is 200-250 words.

*The abstract has now been amended accordingly.*

**Reviewer 1**

This article describes a study exploring the use and potential effectiveness of an electronic questionnaire and automated response to promote help seeking for four risky health behaviors (smoking, alcohol consumption, physical activity, and fruit/vegetable consumption). Among 816 participants included, 602 had at least on risky behavior, and 28% of these had no plan to change this/these behavior(s) and took part in the study. About 2/3 of participants were able to select an applicable item on the tested scale (the Risk Acceptance Ladder - RAL). Also, about 2/3 of participants reported that it was easy to select one option. Participants were then randomized to receive a short message, which was either congruent or incongruent with their RAL response. Compared with the incongruent group, participants allocated to the congruent group had greater odds of clicking on a link to receive further information about this risky behavior. Overall, this study is scientifically sound and was carefully conducted. The reporting is of excellent quality in general. Nevertheless, a few caveats are to be considered to strengthen this manuscript. Those are listed below.

*We thank the reviewer for their helpful comments on our manuscript.*

p.5 lines 118-129, Procedure. This section describes the participants flow. Part of it is redundant with the first section of the results (p. 9-10).

*Thank you for spotting this – we have now shortened the ‘Procedure’ section.*

p.8, line 211, “Free-text responses were coded and summarised in line with standard thematic analysis (17)”. Authors should develop their description of this analysis. Referring to a methods article is not sufficient. Qualitative analysis should be indicated as a sub-heading and details on how it was conducted should be provided. How exactly were data coded? Who did code the responses? Was there double coding? Any triangulation of the data and related coding? Also, authors should discuss the potential limitations of their analytic perspective in the relative discussion section.

*We have now included a ‘Qualitative analysis’ section where we provide more detail on the analysis (p.9):*

*“Free-text responses to the RAL were analysed by CS and LS with inductive thematic analysis (17), which involved generating initial codes and higher-order themes that captured respondents’ underlying beliefs. Responses were coded by CS and double checked by LS, with higher-order themes refined through discussion with RW.”*

The discussion is quite succinct and does not put the findings in perspective with the literature in the field.

*We have now briefly situated our results within the extant literature on p.15 whilst also drawing the reader’s attention to several important limitations on p.16-17 which mean our results should be considered exploratory:*

*“This finding adds to the currently mixed evidence on the effectiveness of health interventions tailored to participants’ motivational stage, assessed at baseline (12–15).”*

*“This study had several limitations. First, as this pilot study was not pre-registered, the results should be considered exploratory. Second, there was a lack of early user involvement in the development of the RAL. As mentioned above, further qualitative work is therefore needed to explore a wider range of RAL items. Third, due to the small sample size, it was not possible to estimate whether the targeted messages were equally effective for all health behaviours. Fourth, although the on-target intervention was found to increase subsequent engagement with health messaging, it is unclear whether this engagement translates to actual behaviour change. Fifth, respondents who selected ‘Other’ on the RAL received off-target messages, with sensitivity analyses conducted to assess whether the direction of the effect remained robust when excluding this group. Although the exclusion of those who selected ‘Other’ did not alter the direction of the effect, the difference no longer reached statistical significance. This may be due to low power for this comparison or be indicative of a potential bias whereby those selecting ‘Other’ were consistently less motivated to change compared with those who were able to select a single ‘rung’ on the RAL. Sixth, our recruitment primarily targeted university students, with the resulting sample being predominantly White, female and relatively young (i.e. an average age of ~30 years). This likely limits the generalisability of the results to the general population without current plans to change the selected health behaviours. Finally, as the study was conducted in 2015, future applications of the RAL may benefit from updating the website design to ensure it aligns with users’ evolving expectations.”*

Discussion, 2nd paragraph. Authors should discuss the small effect size and the low proportion of participants “engaging” further (31%, respectively 19%, accessed a website, but we do not know how far they went on this website).

*Please see our response to the previous comment.*

**Reviewer 2**

This paper is clear and well written. It reports on a potentially useful approach for targeting health behaviour change with patients in time constrained settings.

*We thank the reviewer for their helpful comments on our manuscript.*

Recruitment targeted university students in one avenue, did this limit the demographics of the sample – they were predominantly white, were they also educated, and what are the potential implications of this?

*We have highlighted this as a limitation on p.17:*

*“Sixth, our recruitment primarily targeted university students, with the resulting sample being predominantly White, female and relatively young (i.e. an average age of ~30 years). This likely limits the generalisability of the results to the general population without current plans to change the selected health behaviours.”*

The data is quite old, having been collected in 2015. I’m not sure if that is relevant but it seems a long gestation time for this study.

*The data were collected as part of a student project, which delayed the write up in paper form due to changes in supervision and institutional changes by co-authors. We appreciate that this delay may have impacted current appraisal of the intervention and this has now been highlighted as a limitation on p.17:*

*“Finally, as the study was conducted in 2015, future applications of the RAL may benefit from updating the website design to ensure it aligns with users’ evolving expectations.”*

The measure of ‘excessive alcohol consumption’ seems somewhat crude – drinking every day is not in itself excessive if the daily consumption is limited to less than two standard drinks – perhaps the authors could provide some explanation/justification for the health behaviour measures chosen.

*An explanation has now been added on p.8:*

*“The alcohol item was designed to broadly map onto validated quantity-frequency screening instruments such as the Alcohol Use Disorders Identification Test-Consumption (AUDIT-C) scale, with daily or almost daily drinking classified as excessive alcohol consumption (17,18).”*

The high rate of endorsement of ‘other’ seems to suggest that the RAL is missing important patient beliefs which could be incorporated in an adjusted version. The authors mention possible re-wording but could provide more specific examples of potential future adaptations.

*We have now added a couple of specific examples of potential future adaptations on p.16:*

*“However, some free-text responses were not easily captured by existing RAL items, most notably the inability to change behaviour due to a physical illness or condition (endorsed by a quarter of participants who selected ‘Other’). This previously unaddressed theme relates to physical capability in the COM-B model and an additional item to capture this issue (e.g. “I don't think I can XXXX because I am not physically able to do so” or “I don’t think I can XXXX because it will make me physically uncomfortable in some way”) has the potential to add to the value of the RAL.”*

The authors have conducted appropriate sensitivity analysis to capture the potential bias, but could be more explicit in the discussion in addressing the fact that respondents selecting ‘other’, who all cited negative attitudes towards behaviour change or disbelief of the risks of the behaviour, were allocated to off target messages – this could bias the results in that the motivation levels of these individuals would appear to be lower than that of people who can least appreciate the value of the indicated behaviour change.

*We have reflected on this potential bias on p.16-17:*

*“Fifth, respondents who selected ‘Other’ on the RAL received off-target messages, with sensitivity analyses conducted to assess whether the direction of the effect remained robust when excluding this group. Although the exclusion of those who selected ‘Other’ did not alter the direction of the effect, the difference no longer reached statistical significance. This may be due to low power for this comparison or be indicative of a potential bias whereby those selecting ‘Other’ were consistently less motivated to change compared with those who were able to select a single ‘rung’ on the RAL.”*

The limited nature of outcome assessment should be specifically addressed in the discussion – the impact of targeted messaging is limited to clicks to relevant websites which is an immediate measure only, and does not indicate intentions or actual behaviour change.

*This has been highlighted as a limitation on p.16:*

*“Fourth, although the on-target intervention was found to increase subsequent engagement with health messaging, it is unclear whether this engagement translates to actual behaviour change.”*
