# Supplementary material for: Pilot randomised controlled trial of the Risk Acceptance Ladder (RAL) as a tool for targeting health communications
Source: PLoS One. 2021 Nov 15;16(11):e0259949. doi: 10.1371/journal.pone.0259949 (PMC8592458; doi:10.1371/journal.pone.0259949)
Supplement: Attachment — Submitted filename: Response to Reviewers_R1.docx [file pone.0259949.s003.docx]

**Response to Reviewers**

**Editor**

Our guidelines for data availability (https://journals.plos.org/plosone/s/data-availability) indicate that:

For studies analyzing data collected as part of qualitative research, authors should make excerpts of the transcripts relevant to the study available in an appropriate data repository, within the paper, or upon request if they cannot be shared publicly. If even sharing excerpts would violate the agreement to which the participants consented, authors should explain this restriction and what data they are able to share in their Data Availability Statement.

As your manuscript only contains one quote, we don't think that this has been met. We request that you update your data availability statement and indicate whether the qualitative data can be shared, and where they can be obtained.

*As per our previous Data Availability Statement, we provide quantitative data for this study at* [*https://doi.org/10.6084/m9.figshare.12942053.v1*](https://doi.org/10.6084/m9.figshare.12942053.v1) *. With regards to qualitative data (i.e., open responses) these are not included in the quantitative data set as we did not ask for explicit consent for participants to share these. However, this information is available on request from the corresponding author. The Data Availability Statement has been amended accordingly.*

*Please note that the reference list has been checked, and we can confirm this is complete and correct.*

**Reviewer #1**

Authors adequately addressed reviewers' comments and the current manuscript has been improved and would be ready for publication.

*Many thanks for your support.*
